# Multiomics Unravelling the Mechanisms of Intratumoral Microbiota Influencing Tumorigenesis and Progression in Renal Clear Cell Carcinoma

**DOI:** 10.1111/jcmm.71020

**Published:** 2026-01-07

**Authors:** Keyuan Lou, Jiankun Zhang, Yuanshan Cui

**Affiliations:** ^1^ Department of Urology The Affiliated Yantai Yuhuangding Hospital of Qingdao University Yantai China; ^2^ School of Clinical Medicine Shandong Second Medical University Weifang China; ^3^ Department of Urology Weifang People's Hospital Weifang China; ^4^ Department of Urology Yantai Yuhuangding Hospital Yantai China

To the Editor,

Renal cell carcinoma (RCC) is among the top ten most commonly diagnosed cancers globally, with clear cell renal cell carcinoma (ccRCC) accounting for the majority (70%) of RCC‐related deaths [1]. Recent research has confirmed that the progression of ccRCC is associated with cellular metabolism within the tumour immune microenvironment [2]. Additionally, the intratumoral microbiota plays a pivotal role in gene regulation, oncogenesis, progression and treatment of various cancers, including ccRCC [3]. However, there remains a gap in academic research regarding the presence of intratumoral microbiota in ccRCC and their potential impact on tumorigenesis and progression through the regulation of certain key genes or production of specific metabolites. Therefore, based on existing surgical and immunotherapeutic approaches, exploring the fundamental mechanisms of the interaction between intratumoral microbiota and the tumour microenvironment and identifying key microbiota as potential therapeutic targets, could provide a unique therapeutic strategy for ccRCC.

This study selected 30 pairs (60 samples) of ccRCC tissues and their adjacent normal tissues for microbiota sequencing by 2bRAD‐M. Among these, 20 samples were selected for transcriptome sequencing and 22 samples were subjected to metabolome sequencing. The samples were obtained from 30 patients diagnosed with ccRCC and underwent laparoscopic radical nephrectomy or laparoscopic partial nephrectomy at Yantai Yuhuangding Hospital between February 2022 and December 2023, including 23 males and 7 females. The age range of the male patients was 42 to 70 years, and that of the female patients was 37 to 71 years. The sample donors had no history of infection or antibiotic use within 4 weeks prior to surgery. Samples were rapidly frozen using liquid nitrogen and stored at −80°C until subjected to omics analysis. During the surgical procedure, the sampling, preservation and transportation were strictly conducted under sterile conditions. The tumour staging, grading and immunohistochemical results reported for the samples used in this study were all provided by the Clinical Laboratory of Yantai Yuhuangding Hospital. The sequencing and data analysis in this study were provided by OE Biotech Inc., Shanghai, China. The present study has obtained voluntarily signed informed consent forms from the participants. The protocol of this study was approved by the Ethics Committee of Yantai Yuhuangding Hospital (No. 2024‐194).

The results of microbiota sequencing revealed significant differences in α and β diversity of microorganisms between cancer tissues and adjacent non‐cancer tissues in 23 male and 7 female samples, demonstrating variations in the abundance and distribution of microbial communities between males and females. Postoperative pathological results indicated a notable distinction in α diversity of microbial communities within cancer tissues between 3 samples with vascular tumour thrombus and the remaining 27 samples. Furthermore, tumour stage, serum unsaturated iron‐binding capacity and the positive rates of ki67, PAX‐8, vim, P504S and SDHB in cancer tissues all influenced the β diversity of microorganisms within cancer tissues. We also characterised the differentially abundant microorganisms across different groups, investigated their interaction networks and predicted the functional composition of known microbial genes through Gene Ontology and Kyoto Encyclopedia of Genes and Genomes functional analyses.

Metabolomic sequencing was conducted on 22 samples, revealing separation in principal component analysis (PCA) results of metabolites between cancer tissues and adjacent tissues, as well as when grouped by tumour stage, grade, gender, presence of vascular tumour thrombus and ki67 positivity. These findings suggested metabolite differences among the compared groups. Additionally, we screened for differential metabolites across different groups, identified metabolite‐related signalling pathways and conducted trend analyses of metabolite concentrations over time.

Transcriptomic sequencing was performed on 20 samples to investigate gene expression differences between cancer tissues and adjacent non‐cancer tissues, as well as within groups stratified by tumour stage, grade, gender and ki67 positivity. We sought unique and common differential genes within and between these groups and predicted their associated signalling pathways. Additionally, we conducted transcription factor and target gene prediction analyses, along with protein–protein interaction network analyses, to systematically explore the functions of these differential genes (Figure 1).

**FIGURE 1:**
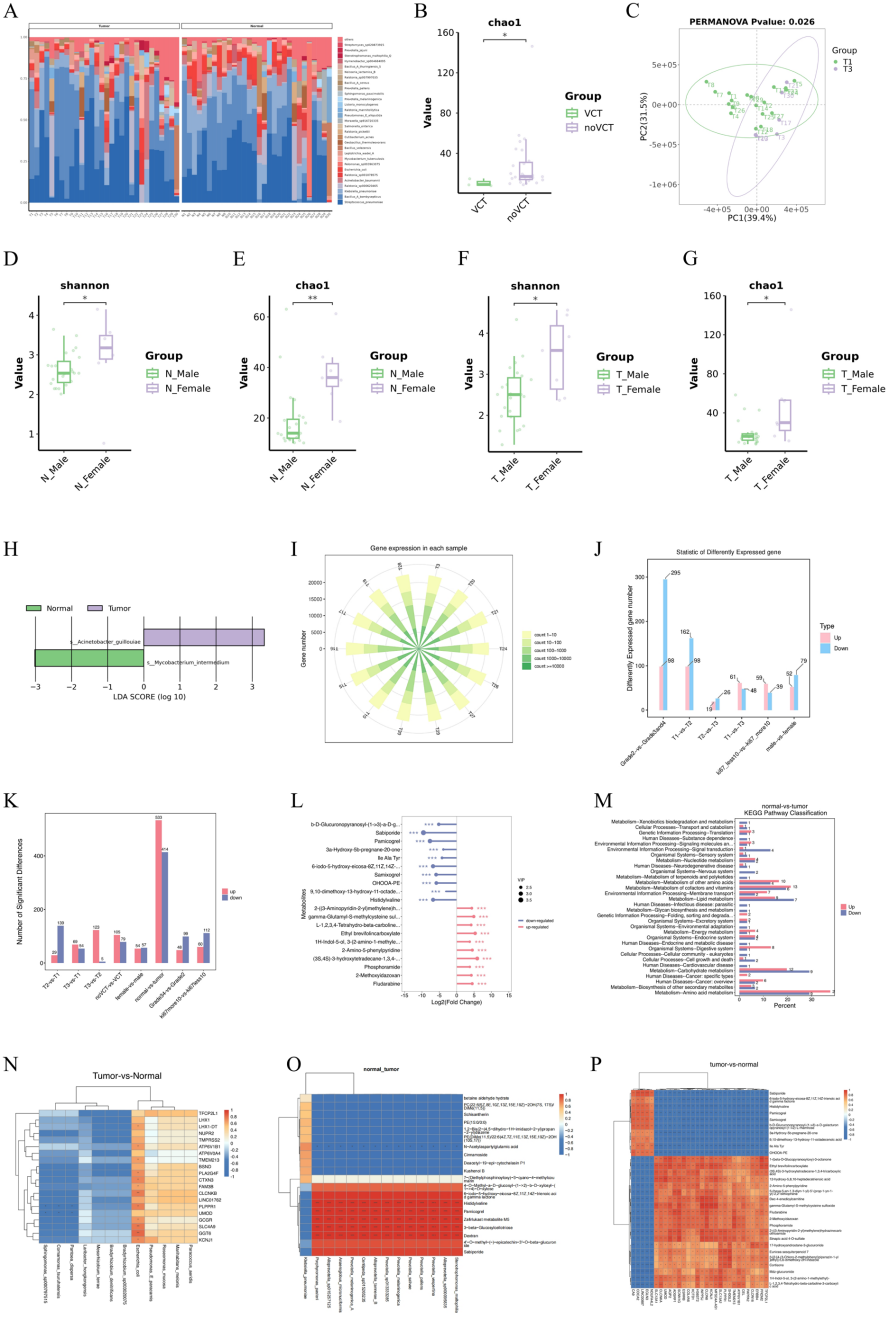
(A) Bar plot of microbial community structure at the species level, showing the top 30 most abundant taxa across samples. (B) Boxplot analysis of alpha diversity. Chao index distributions in cancerous tissues with (VCT) and without vascular cancer thrombus (no VCT) groups, demonstrating significant differences in alpha diversity between the two groups. (C) Beta diversity‐based PCoA analysis of clear cell renal cell carcinoma (ccRCC) tissues at stages T1 and T3. Significant separation of sample distributions along PCoA axes indicates distinct microbial community structures between the two groups. (D) Boxplot analysis of alpha diversity. Distribution of Shannon index in adjacent normal tissues from male patients (N Male) and female patients (N Female), demonstrating significant differences in alpha diversity between the two groups. (E) Boxplot analysis of alpha diversity. Distribution of Chao index in adjacent normal tissues from male patients (N Male) and female patients (N Female), demonstrating significant differences in alpha diversity between the two groups. (F) Boxplot analysis of alpha diversity. Distribution of Shannon index in tumour tissues from male patients (T Male) and adjacent normal tissues from female patients (N Female), demonstrating significant differences in alpha diversity between the two groups. (G) Boxplot analysis of alpha diversity. Distribution of Chao index in tumour tissues from male patients (T Male) and adjacent normal tissues from female patients (N Female), demonstrating significant differences in alpha diversity between the two groups. (H) LEfSe multivariate analysis‐derived differential species score plot between tumour tissues (tumour group) and adjacent normal tissues (normal group). (I) Bar chart of the number of genes detected via transcriptome sequencing. (J) Bar plot of differentially expressed genes (DEGs) across comparison groups. (K) TBar chart of differentially abundant metabolites identified in metabolome sequencing across comparison groups. (L) Lollipop plot of the top 10 upregulated and downregulated metabolites with the smallest *p*‐values between tumour and normal groups. (M) Distribution of upregulated and downregulated differential metabolites at KEGG Level2 between tumour and normal groups. (N) Integrated microbiota‐transcriptome analysis. Correlation heatmap of the top 30 species‐level microbial features (ranked by importance via full feature selection) between tumour and normal groups. (O) Integrated microbiota‐metabolome analysis. Correlation heatmap of the top 20 microbial species (ranked by log2(FoldChange)) and metabolites (ranked by significance) between Tumour and Normal groups at the species level. (P) Integrated transcriptome‐metabolome analysis. Correlation heatmap of the top 30 significantly differentially expressed genes and metabolites (ranked by significance) between tumour and normal groups.

This study aims to explore the differences in renal microbial communities between different genders and investigate whether specific microorganisms influence gene expression by producing certain metabolites, thereby affecting the initiation and progression of ccRCC. Identifying key microorganisms as potential therapeutic targets could be useful in predicting patient prognosis and optimising immune therapeutic responses. If the sample size could be further increased and the number of samples included in each comparison group were unified, the statistical significance would be more meaningful and persuasive.

## Author Contributions

K.L. did the data collection and curation, writing – original draft. J.Z. did the supervision and visualisation, Y.C. did the conceptualisation, funding acquisition and writing – review and editing. All authors have seen and approved the final version of the abstract for publication.

## Funding

This work was supported by Joint Fund of Shandong Natural Science Foundation (Grant ZR2021LSW019), the Taishan Scholars Program of Shandong Province (Grant tsqn202306403).

## Ethics Statement

The protocol of this study was approved by the Ethics Committee of Yantai Yuhuangding Hospital (No. 2024‐194).

## Consent

The authors have nothing to report.

## Conflicts of Interest

The authors declare no conflicts of interest.

## Data Availability

All data generated or analysed during this study are included in this published article.
